# COVID-19 Vaccine Hesitancy Among Older Adults in a Geriatric Centre in Nigeria

**DOI:** 10.7759/cureus.51102

**Published:** 2023-12-26

**Authors:** Lawrence A Adebusoye, Eniola O Cadmus, Oluwagbemiga Oyinlola, Obadare Abiola

**Affiliations:** 1 Geriatrics, Chief Tony Anineh Geriatric Centre, University College Hospital, Ibadan, NGA; 2 Community Medicine, University College Hospital, Ibadan, NGA; 3 Medical Social Services, University College Hospital, Ibadan, NGA; 4 School of Social Work, McGill University, Montreal, CAN

**Keywords:** vaccine hesitancy, covid-19, southwestern nigeria, geriatric patient, older adult

## Abstract

Introduction

Despite the proven efficacy of COVID-19 vaccines, there is a significant level of hesitancy, particularly among the elderly population in Nigeria. The research investigates factors associated with COVID-19 vaccine hesitancy among older persons attending Geriatric Centers in southwestern Nigeria.

Methods

This was a cross-sectional study of 332 older adults (≥60 years). Sociodemographic characteristics, attitudes, beliefs, psychological antecedents, sources of information, and perceived sensitivity to the COVID-19 vaccine were explored. Bivariate and multivariate analyses were performed using the Statistical Package for the Social Sciences (IBM SPSS Statistics for Windows, IBM Corp., Version 27.0, Armonk, NY) at a 0.05 significance level.

Results

The mean age of the respondents was 71.8±7.3 years. The prevalence of COVID-19 vaccine hesitancy was 43.1%. Predictors of COVID-19 hesitancy were not knowing where to get vaccination OR=7.058 (1.745-28.542), did not think vaccines are safe OR=8.767 (2.250-34.159), worries about unforeseen future effects OR=1.111 (1.004-1.227), preference for natural immunity OR=1.170 (1.036-1.321).

Discussion

COVID-19 vaccine hesitancy was high in our study. Our study underscores the importance of community engagement, education, and communication strategies tailored to the needs and perceptions of the older population in Nigeria.

## Introduction

The COVID-19 pandemic has emerged as one of the most significant public health crises of our time, challenging nations across the globe to confront unprecedented uncertainties [1]. In Nigeria, there is an exponential increase in the population of older adults, with a global ranking of 24th among countries, and older adults aged 60 years and above are projected to increase from the current 6.98 million to 25.5 million by the year 2050 [2]. Despite this steady increase in life expectancy and the demographic shift towards an ageing society in Nigeria, there exists the precarity of limited access to geriatric health care services and fragmented infrastructural services [3,4]. While vaccination is still the most effective approach for protecting older adults against COVID-19, hesitancy to be vaccinated remains a complex obstacle hindering efforts to achieve widespread immunization to protect older adults against COVID-19.

The WHO Strategic Advisory Group on Experts (SAGE) on Immunization defined vaccine hesitancy as "a delay in acceptance or refusal of vaccination despite the availability of vaccination services" [5]. Hesitancy can be complex and varies across place and time [4,6,7]. Vaccines are one of the most cost-effective public health measures to prevent infectious diseases, estimated to save about 2.5 million lives annually [8,9]. The benefits of vaccines often outweigh the risks. The WHO recommends a "life-course" approach to vaccination [8,9]. The "life-course" approach to vaccination encourages equity in vaccine access so that every age group, including the elderly, benefits from the protection offered by vaccines [8,9]. The routine vaccines recommended for adults 60 and older include COVID-19, influenza, pneumococcal, tetanus, diphtheria, pertussis, and varicella zoster vaccine [10]. There are specific vaccination programmes against influenza and *Streptococcus pneumoniae* for older adults with a background disease and heterogeneous age limits between ≥ 50 and ≥65 years [11,12]. The choice of whether or not to receive a vaccination is one that many older adults make with ease: either they decide to obtain every recommended vaccination, or they completely refuse vaccinations. Others, however, decide differently, considering each situation individually. This intermediate class of individuals, who pick and choose which vaccinations they embrace, is referred to by experts as "vaccine-hesitant" [13,14].

Extant literature has reported how the historical experiences of COVID-19 vaccination campaigns, access to information about the vaccine, and the dissemination of misinformation all intertwine to shape vaccine decision-making among older adults due to the sociopolitical situations of a country [3,4,9,15,16]. These studies highlight how older adults' vaccine acceptance or reluctance to take COVID-19 vaccines is a concern and a humanitarian crisis. Still, vaccine hesitancy in older adults cannot be viewed in isolation. Still, it should be analyzed within a broader context of those attending a geriatric centre in a large country like Nigeria, where infrastructural resources and support programs are unavailable. Furthermore, the 5As practical taxonomy for the determinants of vaccine uptake explored the determinants of vaccine hesitancy, including acceptability, accessibility, affordability, awareness, and activation; these are the myriad possible root causes of vaccine uptake [4,17], the tool was utilized within the context of the COVID-19 vaccine in the African region [4].

Although empirical evidence focusing on vaccine hesitancy among older adults during a pandemic is scarce and unclear, this study is an important step toward starting a conversation toward vaccination of older adults beyond the scope of COVID-19 across the African region. Therefore, understanding the complexities of vaccine hesitancy among older adults in Nigeria will lay the groundwork for designing evidence-based public health interventions that can be applied in future vaccination campaigns and other health initiatives. Hence, this study aimed to investigate the factors associated with COVID-19 vaccine hesitancy among older adults attending geriatric centres in Nigeria.

## Materials and methods

Study design

This cross-sectional hospital-based study investigated factors associated with COVID-19 vaccine hesitancy among older adults attending geriatric centres in Nigeria. This was conducted among older adults attending the Chief Tony Anenih Geriatric Centre (CTAGC), University College Hospital (UCH), Ibadan. Ibadan is the capital city of Oyo State in the southwestern area of Nigeria and has a population of 3.6 million inhabitants, while Oyo State has 5.6 million people [4]. The CTAGC is a purpose-built centre, that was established on November 17, 2012, to provide holistic care to older patients coming to UCH. CTAGC is the pioneer geriatric centre in Nigeria and provides both in-patient and out-patient services.

Study population

Male and female patients aged 60 years and above who presented at the CTAGC, UCH, were recruited into the study between January and March 2023. The age of the respondents was determined by asking them. Similar to other research, for those unable to provide the necessary information, their ages were estimated using historical events, the age at marriage, and the age of their first child [18-20]. Those who did not consent or were too ill to participate were excluded.

Sample size determination

The sample size was determined using Leslie and Kish's calculation for a single proportion. The prevalence of vaccine hesitancy (68.5%) in the six geopolitical zones of Nigeria [21] and a precision of 5% were used in calculating the sample size to arrive at 332 respondents. A systematic sampling method was employed. Based on the expected 630 patients during the three months of the study and a sample size of 332, a sample interval of 1.9 (630/332) ≈ 2 was used. Thus, one in every two older patients presenting at the CTAGC clinic was recruited.

Procedure

A semi-structured interviewer-administered questionnaire was pre-tested before the actual study was used. The respondents' demographic characteristics, such as age, sex, ethnicity, religion, marital status, number of children, educational level, income, occupational status, living arrangement, lifestyle habits, and financial and social support were obtained.

Vaccine hesitancy was determined by asking the respondents firstly "Have you had COVID-19 vaccines? [10]. The possible answers are "Yes and No". For those who answered "No" (had not been vaccinated for COVID-19) would be asked again "Would you like to take the COVID-19 vaccine if it was available now?" The response options included "Yes, No and Undecided". The respondents were divided into two groups ("Vaccine Hesitancy" and "Not Vaccine Hesitancy") [22]. Those categorized as having "COVID-19 Vaccine Hesitancy" were respondents who were undecided and unwilling to get vaccinated. Those who have had the COVID-19 vaccine and would like to be vaccinated were classified as "Not COVID-19 Vaccine Hesitancy".

Determinants of vaccine hesitancy

The determinants of vaccine hesitancy were assessed using validated tools.

The psychological antecedents to vaccination were assessed with the “5C scale” [23]. It is comprised of five three-item subscales to measure: Confidence (e.g., "I am confident that public authorities decide in the best interest of the community"), complacency (e.g., "vaccination is unnecessary because vaccine-preventable diseases are not common anymore"), Constraints (e.g., "it is inconvenient to receive vaccinations"), Calculation (e.g., "for each and every vaccination, I closely consider whether it is useful for me"), and Collective Responsibility (e.g., "vaccination is a collective action to prevent the spread of diseases"). Responses are measured on a seven-point Likert scale (1 = strongly disagree, 7 = strongly agree) and scored by calculating the mean score for each subscale (score range 1-7). Higher confidence and collective responsibility scores indicate enablers of vaccination, while higher complacency, calculation, and constraint scores indicate more individual barriers to vaccination [23].

The general vaccination attitudes and predicted vaccination behaviour were measured with the Vaccine Attitude Examination (VAX) scale. The 12-item VAX scale is short, has high internal consistency reliability, and comprises four distinct but correlated factors [11,24]. The scale contains four three-item subscales: Mistrust of Vaccine Benefits, Worries about Unforeseen Future Effects, Concerns about Commercial Profiteering, and Preference for Natural Immunity [11,24]. All items were measured on a six-point scale. Each item is scored on a scale of "1 = strongly disagree" to "6 = strongly agree". and scored by calculating the mean scores for each subscale as well as a mean total score (range 1-6). Lower scores indicate more positive vaccination views, while higher scores represent more negative views [11,24]. The VAX scales were further sub-categorized based on item number: items #1-3 relate to mistrust of vaccine benefits, #4-6 to worries over unforeseen future effects, #7-9 to concerns about commercial profits, #10-12 to the preference for natural immunity [13,24].

The 5-item perceived sensitivity to medicines (PSM) scale was used to assess the respondents' possible reaction to a vaccine [25]. A person's beliefs about their sensitivity to medicines affect the perception and reporting of medication side effects. Thus, it was predicted that a high score on the PSM scale would predict a greater number of symptoms following vaccination. Responses are scored on a 5-point Likert-type scale. Individual item scores are summed to provide a total PSM score ranging from 5 to 25. High scores indicate high perceived sensitivity to potential adverse effects of medicines [25].

Consent for the study

Approval for the study was obtained from the University of Ibadan Institutional Ethical Review Board (UI/EC/22/0351). Informed consent of each respondent will be obtained before administering the questionnaire.

Data analysis

The administered questionnaires were cleaned and analyzed using the Statistical Package for the Social Sciences (IBM SPSS Statistics for Windows, IBM Corp., Version 21, Armonk, NY). Descriptive statistics were used to describe the socio-demographic characteristics of the respondents. Chi-square statistics were used to assess the association between categorical variables and Student’s t-test for the continuous variables. Logistic regression explored the relationship between significant variables and COVID-19 vaccine hesitancy. The level of significance was set at p ≤ 0.05.

## Results

We had a 96% response rate from the questionnaire we distributed to the participants. There were 184 (55.4%) female respondents, and the mean age was 71.8 ± 7.3 years. Their monthly income was 34,000 (IQR 20,000-65,000) Naira. Most respondents were married 233 (70.2%) and 255 (76.8%) had formal education. Sixteen (4.8%) respondents were living alone, 42 (12.7%) were self-supporting financially, and 322 (97.0%) used the clinic as the source of health treatment in the past year (Table 1).

**Table 1:** Baseline characteristics of the respondents (N = 332) The data has been represented as N is considered the number of participants, and % is the percentage of responses received from the participants.

Variables	N	Percentage
Sex		
Male	148	44.6
Female	184	55.4
Age groups (years)		
60-64years	52	15.7
65-69years	81	24.4
70-74years	103	31.0
75-79years	48	14.5
80 years and above	48	14.5
Marital status		
Married	233	70.2
Widowed	96	28.9
Separated	3	0.9
Highest educational attainment		
No formal	77	23.2
Primary	86	25.9
Secondary	57	17.2
Tertiary	112	33.7
Living arrangement		
Alone	16	4.8
With spouse only	192	57.8
With children/grandchildren	109	32.8
With relatives and friends	15	4.5
Financial support		
Self-supporting	42	12.7
Spouse only	16	4.8
Children/grandchildren	272	81.9
Relative and friend	2	0.6
Social support		
Self-supporting	10	3.0
Spouse only	55	16.6
Children/grandchildren	250	75.3
Relative and friend	17	5.1
Source of health treatment in the past one year		
Clinic	322	97.0
Chemist	4	1.2
Traditional settings	4	1.2
Self-medication	2	0.6
Self-rated physical activity		
Not active	19	5.7
Moderately active	248	74.7
Very active	65	19.6

**Figure 1:**
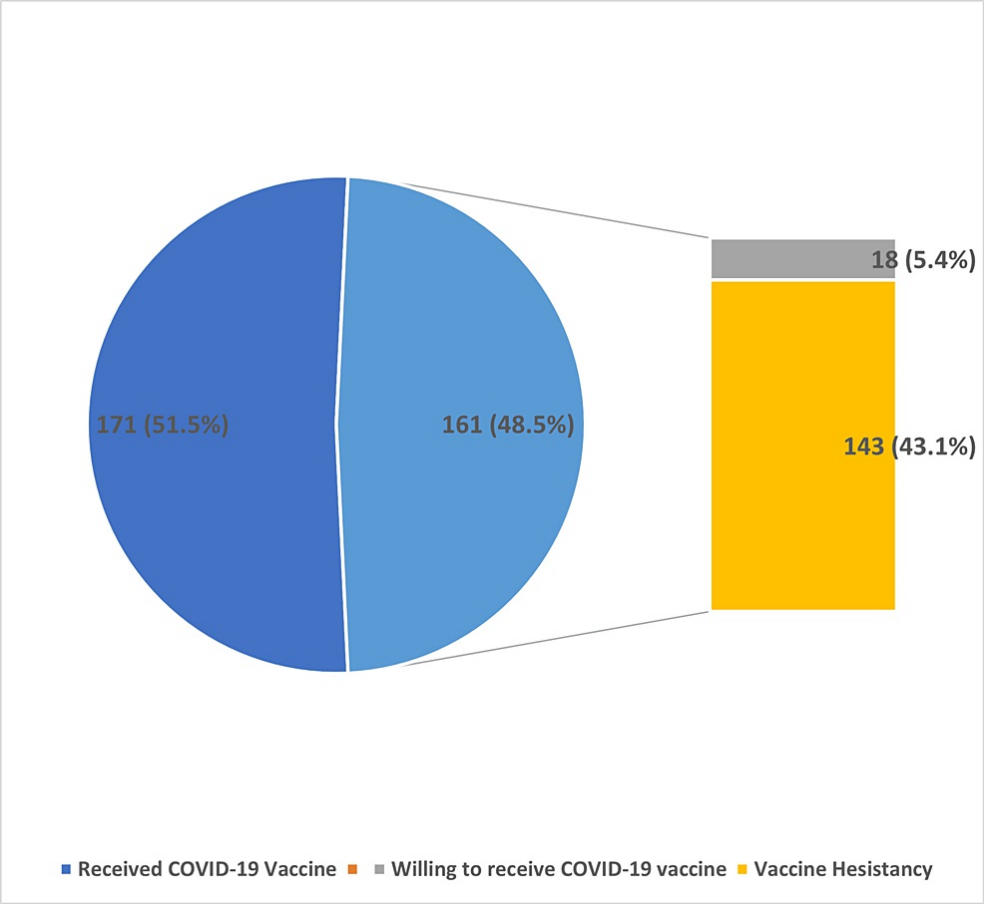
COVID-19 vaccine hesitancy among older persons

One hundred and seventy-one (51.5%) respondents had taken the COVID-19 vaccine, and they were categorized into “Not Vaccine Hesitancy”. The number of older persons who would prefer to get vaccinated against COVID-19 if it was available now was 18 (5.4%) among those who did not receive the COVID-19 vaccination (n = 161). As a result, the overall number of "Not Vaccine Hesitancy" (had COVID-19 vaccination = 171 and would prefer to get vaccinated for COVID-19 if it were available today = 18) was 189 (56.9%). Those who reported being hesitant to be vaccinated (n = 98, 29.5%) or unsure about taking the COVID-19 vaccine (n = 45, 13.6%) were classified as having "vaccine hesitancy" 143 of 332 (43.1%) (Figure 1).

The relationship between sociodemographic factors and COVID-19 vaccine hesitancy among older persons indicated that having formal education (p = 0.010), earning above the minimal wage of 30,000 Naira per month (p <0.0001), and being self-supportive financially (p = 0.002) were significantly associated with vaccine hesitancy (Table 2).

**Table 2:** Sociodemographic factors and COVID-19 vaccine hesitancy The data has been represented as N, meaning the number of participants, % as a percentage score, χ2 as the chi-squared test is a statistical test used in the analysis of contingency tables, and the p-value considered significant (p < 0.05).

	Vaccine Hesitancy			
Variables	Yes = 143 n (%)	No = 189 n (%)	χ^2^	p
Sex				
Male	64 (43.2)	84 (56.8)	0.003	0.955
Female	79 (42.9)	105 (57.1)		
Age Groups (Years)				
60-69 years	51 (38.3)	82 (61.7)	3.126	0.206
70-79 years	73 (48.3)	78 (51.7)		
≥80 years	19 (39.6)	29 (60.4)		
Marital Status				
Currently married	101 (43.3)	132 (56.7)	0.024	0.876
Not currently married	42 (42.4)	57 (57.6)		
Highest Educational attainment				
No formal education	43 (55.8)	34 (44.2)	6.669	0.010*
Had formal education	100 (39.2)	155 (60.8)		
Religion				
Christianity	99 (40.6)	145 (59.4)	2.344	0.126
Islam	44 (50.0)	44 (50.0)		
Occupational status				
Retired	108 (42.7)	145 (57.3)	0.064	0.800
Not retired	35 (44.3)	44 (55.7)		
Monthly Income (30,000 Naira per month)				
Above the minimum wage	56 (32.6)	116 (67.4)	16.091	<0.0001*
Below the minimum wage	87 (54.4)	73 (45.6)		
Living arrangement				
Alone	5 (31.2)	11 (68.8)	0.958	0.328
With others (Spouse, children, relatives)	138 (43.7)	178 (56.3)		
Financial support				
Self-supporting	9 (21.4)	33 (78.6)	9.186	0.002*
By others (Spouse, children, relatives)	134 (46.2)	156 (53.8)		
Social support				
Self-supporting	4 (40.0)	6 (60.0)	0.040	0.842
By others (Spouse, children, relatives)	139 (43.2)	183 (56.8)		

Attitude, psychological antecedents, and perceived sensitivity to vaccination and COVID-19 vaccine hesitancy are described in Table 3. The respondents’ general attitude to vaccination revealed that the mistrust of vaccine benefits (p <0.0001) and worries over unforeseen future effects of vaccines were the significant factors associated with COVID-19 vaccine hesitancy. Similarly, the psychological antecedents to vaccination of the respondents showed that the lack of confidence in vaccination (p <0.0001), calculation (p = 0.046), and collective responsibility (p <0.0001) were the significant factors in COVID-19 vaccine hesitancy. COVID-19 vaccine hesitancy was significantly associated with respondents’ perceived sensitivity to vaccines, especially in those who stated that their bodies were very sensitive to medicines (p = 0.022) and had a bad reaction to medicines (p = 0.049) (Table 3).

**Table 3:** Attitude, psychological antecedents, and perceived sensitivity to vaccination and COVID-19 vaccine hesitancy The data has been represented as N, meaning the number of participants, X is considered the mean, SD is the standard deviation, t is considered the t-test score, and the p-value is considered significant (p < 0.01).

	Vaccine Hesitancy			
Variables	YES = 143 X ± SD	No = 189 X ± SD	t	p-value
Vaccine Attitude Examination				
Mistrust of Vaccine benefits	13.57 ± 5.41	15.80 ± 3.36	-4.392	<0.0001*
Worries over unforeseen future effects	8.31 ± 5.49	9.53 ± 3.91	-2.271	0.024*
Concern about commercial profiteering	7.11 ± 5.03	6.66 ± 4.06	0.873	0.383
Preference for Natural Immunity	6.13 ± 4.45	6.89 ± 3.92	-1.668	0.096
Overall	35.11 ± 10.92	38.88 ± 9.03	-3.353	0.001*
Psychological antecedents to vaccination				
Confidence	15.07 ± 5.95	18.79 ± 3.21	-6.777	<0.0001*
Complacency	7.47 ± 5.49	6.88 ± 4.06	1.072	0.285
Constraints	5.76 ± 4.51	5.80 ± 3.36	-0.093	0.926
Calculation	16.66 ± 5.26	17.75 ± 4.44	-2.005	0.046*
Collective responsibility	9.18 ± 4.66	10.99 ± 4.27	-3.672	<0.0001*
Perceived sensitivity to vaccine				
My body is very sensitive to medicines.	1.47 ± 1.11	1.78 ± 1.30	-2.308	0.022*
My body overreacts to medicines.	1.34 ± 0.97	1.46 ± 1.30	-1.140	0.255
I usually have stronger reactions to medicines than most people.	1.32 ± 0.90	1.39 ± 0.88	-0.682	0.496
I have had a bad reaction to medicines in the past.	1.19 ± 0.77	1.38 ± 0.93	-1.976	0.049*
Even very small amounts of medicines can upset my body	1.25 ± 0.85	1.34 ± 0.93	-0.987	0.325
Total score	6.57 ± 4.12	7.34 ± 4.25	-1.661	0.098

Significantly, respondents who think that vaccines are not needed (p <0.0001), who did not know where to get a vaccination (p <0.0001), and who did not get good/reliable information on vaccines (p <0.0001), who have heard or read negative things about vaccine (p = 0.028), think that vaccine was not effective (p <0.0001), and safe (p <0.0001) reported COVID-19 vaccine hesitancy (Table 4).

**Table 4:** Beliefs about vaccine and COVID-19 vaccine hesitancy of older adults The data has been represented as N, meaning the number of participants, % as a percentage score, χ2 as the chi-squared test is a statistical test used in the analysis of contingency tables, and the p-value considered significant (p < 0.05).

Belief about Vaccine and COVID-19 Vaccine Hesitancy	Response	COVID-19 Vaccine Hesitancy		Total N (%)	χ^2^	p
		YES = 171 n (%)	No = 161 n (%)			
Do you think vaccines are needed?	Yes	112 (37.7)	185 (62.3)	297 (100.0)	33.022	<0.0001*
	No	31 (88.6)	4 (11.4)	35 (100.0)		
Do you know where to get a vaccination?	Yes	98 (35.5)	178 (64.5)	276 (100.0)	38.191	<0.0001*
	No	45 (80.4)	11 (19.6)	56 (100.0)		
Do you know where to get good/reliable information on vaccines?	Yes	95 (36.4)	166 (63.6)	261 (100.0)	22.169	<0.0001*
	No	48 (67.6)	23 (32.4)	71 (100.0)		
Have you heard or read negative things about vaccine?	Yes	59 (36.9)	101 (63.1)	160 (100.0)	4.837	0.028*
	No	84 (48.8)	88 (51.2)	172 (100.0)		
Do you think vaccines are effective?	Yes	102 (36.3)	179 (63.7)	281 (100.0)	34.228	<0.0001*
	No	41 (80.4)	10 (19.6)	51 (100.0)		
Do you think vaccines are safe?	Yes	94 (35.2)	173 (64.8)	267 (100.0)	34.416	<0.0001*
	No	49 (75.4)	16 (24.6)	65 (100.0)		
Has someone told you that the vaccines are not safe?	Yes	50 (40.7)	73 (59.3)	123 (100.0)	0.467	0.494
	No	93 (44.5)	116 (55.5)	209 (100.0)		
Have you had a bad experience with a previous vaccinator/health clinic?	Yes	10 (33.3)	20 (66.7)	30 (100.0)	1.276	0.259
	No	133 (44.0)	169 (56.0)	302 (100.0)		
Have you had a bad experience or reaction to previous vaccination?	Yes	8 (34.8)	15 (65.2)	23 (100.0)	0.693	0.405
	No	135 (43.7)	174 (56.3)	309 (100.0)		
Has someone told you that they had a bad reaction to vaccines?	Yes	22 (44.0)	28 (56.0)	50 (100.0)	0.021	0.886
	No	121 (42.9)	161 (57.1)	282 (100.0)		
Do you have a fear of needles?	Yes	44 (46.3)	51 (53.7)	95 (100.0)	0.571	0.450
	No	99 (41.8)	138 (58.2)	237 (100.0)		
Do you find it difficult to leave other work?	Yes	27 (36.5)	47 (63.5)	74 (100.0)	1.684	0.194
	No	116 (45.0)	142 (55.0)	258 (100.0)		
Do your religious beliefs prevent vaccination?	Yes	4 (33.3)	8 (66.7)	12 (100.0)	0.482	0.488
	No	139 (43.4)	181 (56.6)	320 (100.0)		
Do you have other beliefs/traditional medicine that prevent vaccination?	Yes	5 (62.5)	3 (37.5)	8 (100.0)	1.267	0.261
	No	138 (42.6)	186 (57.4)	324 (100.0)		

The respondents’ sources of information were used by the COVID-19 vaccine hesitancy. Comparatively, there were higher proportions of respondents who did not receive than those who received information on COVID-19 vaccination from the television (62.5% vs 41.1%, p <0.0001), printed and electronic newspaper (46.7% vs 29.6%, p = 0.010), and face-to-face communication (55.5% vs 16.2%, p <0.0001) who reported significant vaccine hesitancy (Table 5).

**Table 5:** Respondents’ sources of information and COVID-19 vaccine hesitancy The data has been represented as N, meaning the number of participants, % as a percentage score, χ2 as the chi-squared test is a statistical test used in the analysis of contingency tables, and the p-value considered significant (p < 0.05).

Sources of information		COVID-19 Vaccine Hesitancy		Total N (%)	χ^2^	p
		Yes = 143 n (%)	No = 189 n (%)			
Social media	Yes	79 (43.9)	101 (56.1)	180 (100.0)	0.107	0.744
	No	64 (42.1)	88 (57.9)	152 (100.0)		
Television	Yes	123 (41.0)	177 (59.0)	300 (100.0)	5.451	0.020*
	No	20 (62.5)	12 (37.5)	32 (100.0)		
Radio	Yes	136 (42.8)	182 (57.2)	318 (100.0)	0.286	0.593
	No	7 (50.0)	7 (50.0)	14 (100.0)		
Printed and electronic newspapers	Yes	21 (29.6)	50 (70.4)	71 (100.0)	6.708	0.010*
	No	122 (46.7)	139 (53.3)	261 (100.0)		
Face-to-face communication	Yes	17 (16.2)	88 (83.8)	105 (100.0)	45.258	<0.0001*
	No	126 (55.5)	101 (44.5)	227 (100.0)		
Official government website	Yes	11 (28.9)	27 (71.1)	38 (100.0)	3.492	0.062
	No	132 (44.9)	162 (55.1)	294 (100.0)		

Logistic regression analysis was carried out on significant variables associated with COVID-19 vaccine hesitancy (Table 6). The logistic model was statistically significant, χ2 (20) = 185.317, p < 0.0001. The model explained 57.6% (Nagelkerke R2) of the variance in COVID-19 vaccine hesitancy and correctly classified 81.6% of cases. The most significant predictor of COVID-19 vaccine hesitancy is not knowing where to get vaccinated (OR = 7.058 95% CI = 1.745-28.542). This is followed by not believing the COVID-19 vaccine is safe (OR = 8.767 95% CI = 2.250-34.159), living below the minimum wage (OR = 2.201 95% CI = 1.156-4.189), concerns about unanticipated side effects (OR = 1.111 95% CI = 1.004-1.227), preference for natural immunity (OR = 1.170 95% 1.036-1.321), decreased confidence in vaccination (OR = 0.778 95% CI = 0.684-0.884), and finally not having face-to-face communication as a source of information (OR = 8.742 95% CI = 3.368-22.690) (Table 6).

**Table 6:** Predictors of COVID-19 vaccine hesitancy The data has been represented as N, meaning the number of participants, % as a percentage score, β is considered, β is the probability that we would accept the null hypothesis even if the alternative hypothesis is true, the OR is considered to report the strength of association, 95% CI was considered the confidence interval that there are 5% chances of being wrong, and the p-value considered significant (p < 0.05).

Predictors of COVID-19 Vaccine Hesitancy	β	p-value	Odds Ratio	95% CI for OR	
				Lower	Upper
Beliefs about vaccine					
Did not think vaccines were needed	0.708	0.426	2.030	0.356	11.578
Did not know where to get the vaccination	1.954	0.006*	7.058	1.745	28.542
Did not know where to get good /reliable information on vaccines	-1.327	0.056	0.265	0.068	1.036
Have not heard or read negative things about vaccine	-0.239	0.486	0.787	0.401	1.544
Did not think vaccine was effective	-0.391	0.590	0.676	0.163	2.804
Did not think vaccines are safe	2.171	0.002*	8.767	2.250	34.159
Sociodemographic characteristics					
Had no formal education	-0.483	0.196	0.617	0.297	1.282
Earned below the minimum wage	0.789	0.016*	2.201	1.156	4.189
Self-supporting financially	-0.899	0.141	0.407	0.123	1.349
Vaccine attitude examination					
Mistrust of vaccine benefits	-0.046	0.462	0.955	0.845	1.080
Worries over unforeseen future effects	0.104	0.041*	1.111	1.004	1.227
Preference for natural immunity	0.157	0.011*	1.170	1.036	1.321
Psychological antecedents to vaccination (5Cs)					
Confidence	-0.251	<0.0001*	0.778	0.684	0.884
Calculation	-0.024	0.525	0.976	0.905	1.052
Collective responsibility	-0.088	0.052	0.916	0.838	1.001
Perceived sensitivity to vaccine					
My body is very sensitive to medicines	-0.067	0.686	0.935	0.677	1.292
I have had a bad reaction to medicines in the past	0.066	0.776	1.069	0.677	1.688
Sources of information					
Television	-0.073	0.914	0.929	0.247	3.495
Printed and electronic newspapers	-0.286	0.597	0.751	0.260	2.172
Face-to-face communication	2.168	<0.0001*	8.742	3.368	22.690
Constant	1.661	0.399	5.266		

## Discussion

The focus of this study is to investigate factors influencing COVID-19 vaccine hesitancy among older persons attending a Geriatric centre in the southwestern region of Nigeria. The mean age of the older adults was above 70 years, consistent with previous findings on the average age of older adults attending a geriatric centre in Nigeria [18,26]. There was also a predominance of female respondents who outnumbered their male counterparts by a ratio of 1.7 to 1. This might be attributed to the reported life expectancy higher for women than men in Nigeria [25,26]. During this study, Nigerian women's life expectancy was 55.8 years compared to 52.9 years [15]. In addition, women visit clinics more frequently than men. Furthermore, a preponderance of the older adults who participated in this study are financially self-supporting. This can be connected with the prevailing concerns of older adults in peri-urban cities like Ibadan, where access to the government’s financial resources is scarce, thus have to rely on their finances coming from their savings [21,27].

Over half of the participants have taken the COVID-19 vaccine and were categorized as not hesitant. This indicated a successful public health effort and a positive step toward controlling the pandemic. However, maintaining and increasing this vaccine acceptance rate requires ongoing collaboration among healthcare providers, government agencies, researchers, and the community [3,6]. Although a few of the older adults who did not take the COVID-19 vaccine were interested in being vaccinated against COVID-19, even if they have access and it is available for them to take. This is connected to the attitudes of older adults, which could be ascribed to previous vaccination experiences, which are considered barriers; for example, during the vaccination boycott in 2003, there was misinformation that taking a vaccine could cause cancer and HIV/AIDS [18,28]. There was scepticism about the rapid vaccine development process or uncertainty about potential side effects [4,23,29]. One intriguing finding is that some older adults who have access and availability to the COVID-19 vaccine still express hesitation. This observation raises questions about the nature of the barriers that these individuals perceive and whether their concerns are rooted in misinformation or valid considerations [28,24,15]. These findings highlight the need for a combination of targeted communication strategies, healthcare provider engagement, cultural sensitivity, and ongoing public health campaigns that can contribute to building trust and fostering vaccine acceptance among this population.

The findings indicated that having no formal education, earnings below the Nigerian minimum wage of 30,000 Naira ($40) per month and being financially and socially supported by others were significantly associated with COVID-19 vaccine hesitancy among the older adults in the geriatric centre. The association between having no formal education and vaccine hesitancy explains why previous studies suggested considering socio-cultural and contextual factors when addressing vaccine acceptance among older adults [15,26]. In addition, the link between lower education levels and heightened vaccine hesitancy aligns with broader patterns observed in vaccine hesitancy empirical evidence, where individuals with limited access to information and education are more susceptible to misinformation and distrust [15,26]. This may be because most older adults earning above minimum wage in Nigeria are either engaged in personal business or receive benefits from the government; hence, the findings could be ascribed to relatively better access to information [3,9] combined with their increased vulnerability to the virus as they engaged in their daily economic activities [15]; Babatope et al. (2023) which in turn contributed to their greater willingness to get vaccinated [4].

The findings indicated that older adults’ mistrust of the vaccine benefits and worries over unforeseen future effects were the significant factors associated with COVID-19 vaccine hesitancy. Similar studies from developed countries like the United States and Canada indicated that, historically, vaccine mistrust could stem from various sources, including historical medical injustices, misinformation, lack of transparency, and concerns about the motives behind vaccine distribution [25]. However, developing countries like Nigeria have reported mistrust of government vaccination programmes [24] and specific cultural beliefs or practices that influence their perceptions of COVID-19 vaccines [4]. In addition, the respondents’ lack of confidence in vaccination could be attributed to the concerns about potential side effects and the belief that the COVID-19 vaccine may exacerbate their health issues since multimorbidity is often present in most older persons [22,30]. COVID-19 vaccine hesitancy was significantly associated with respondents’ perceived sensitivity to vaccines, especially those who reported that their bodies were susceptible to medicines. This finding is consistent with an extensive survey from five sub-Saharan African countries where older adults reported being scared of the adverse reaction and body sensitivity to the COVID-19 vaccine [23,31].

Significantly, a higher proportion of respondents who think that the vaccines are unnecessary had COVID-19 vaccine hesitancy. This is unsurprising as diverse factors, including personal belief and misunderstanding, influence the choice to receive vaccination [26]. Like the younger age group, older adults can be exposed to misinformation, false claims, and conspiracy theories circulating online or through social networks. Evidence from the peri-urban community indicated that misinformation led to scepticism about the necessity and safety of vaccines [15,5]. Evidence from rural Nigeria indicated that some individuals believe they already have a natural immunity to COVID-19 due to previous exposure or an assumption that their age makes them less susceptible to the virus [3]. Therefore, addressing vaccine hesitancy among older adults in a multicultural setting like Nigeria requires a multi-faceted approach involving education, clear communication of vaccine benefits and risks, dispelling myths and misconceptions, and building trust in healthcare institutions and providers.

Within the context of sources of information about COVID-19 vaccine hesitancy, we found that higher proportions of respondents who had vaccine hesitancy did not receive COVID-19 vaccine information from television, newspapers, or face-to-face communication compared to those who had access to these media. This is similar to the reported importance of television in accessing COVID-19 information [11]. Furthermore, the study reported that access to printed and electronic newspapers significantly influences vaccine hesitancy among older adults [9]. Not surprisingly, obtaining COVID-19 information from social media and the official government website was not significantly associated with vaccine hesitancy as older adults are less familiar with and/or lack access to the internet and thus rely more on television and printed newspapers as sources of information [28]. Newspapers often feature opinions and insights from experts in the medical field, and statements from credible medical professionals endorsing the safety and efficacy of vaccines can positively impact vaccine acceptance [15]. Our study noted the association between "face-to-face communication" and vaccine hesitancy. Extant literature indicates that older adults often rely on health information from family members, friends, community leaders, and healthcare professionals [28]. Furthermore, community leaders hold sway over public opinion. When these respected leaders endorse vaccines and encourage their followers to do so in "face-to-face communication", it always positively impacts vaccine acceptance [4,31]. Nigeria is linguistically diverse, with a low literacy rate, especially among older adults. Using "face-to-face communication" could break the language barrier as information is conveyed adequately in the local dialects.

The factors which predict COVID-19 vaccine hesitancy were not knowing where to get vaccination (odds ratio x 7.1), did not think vaccines are safe (odds ratio x 8.8), living below the Nigerian monthly minimum wage (odds ratio x 2.2), worries about unforeseen future effects of COVID-19 vaccine (odds ratio x 1.1), having a preference for natural immunity (odds ratio x 1.2), having reduced confidence in COVID-19 vaccine (odds ratio x 0.78), and not having a face-to-face communication as a source of COVID-19 vaccine information (odds ratio x 8.7). In terms of not knowing where to get the vaccination, across African regions, access to COVID-19 vaccination is one of the most common problems of COVID-19 vaccine hesitancy among older adults [4,15,26]. This was due to logistical challenges relating to the vaccine distribution across large and diverse countries like Nigeria, especially those residing in rural communities [4]. Additionally, transportation infrastructure and cold chain requirements (some vaccines require specific temperature conditions) could have affected equitable distribution [15]. Countries like Nigeria had issues relating to online registration and appointment scheduling, which were common methods for COVID-19 vaccine distribution [19,29]. Older adults who are less familiar with digital technology or lack access to the internet might have struggled to register for vaccinations, causing further barriers to access and vaccine hesitancy [32].

## Conclusions

About a third of older persons in our setting had COVID-19 vaccine hesitancy which was unacceptably high because COVID-19 morbidity and mortality was highest among them. Our study highlighted the myriad of factors such as the general vaccination attitudes and behaviour, psychological antecedents to vaccination, perceived sensitivity to medicines, and sources of information on the COVID-19 vaccine which if addressed could mitigate the hesitancy to COVID-19 vaccination and subsequently other vaccines recommended for older adults. The limitation of this study was that vaccine hesitancy was determined by self-report which could be susceptible to bias. Also, the findings might be difficult to generalise because it is a hospital-based study.

## Appendices

**Table 7:** Beliefs about vaccine and COVID-19 vaccine hesitancy

	Yes	No	
			Do you think vaccines are needed?			
Do you know where to get a vaccination?			
Do you know where to get good/reliable information on vaccines?			
Have you heard or read negative things about vaccine?			
Do you think vaccines are effective?			
Do you think vaccines are safe?			
Has someone told you that the vaccines are not safe?			
Have you had a bad experience with a previous vaccinator/health clinic?			
Have you had a bad experience or reaction to previous vaccination?			
Has someone told you that they had a bad reaction to vaccines?			
Do you have a fear of needles?			
Do you find it difficult to leave other work?			
Do your religious beliefs prevent vaccination?			
Do you have other beliefs/traditional medicine that prevent vaccination?			

**Table 8:** Determinants of vaccine hesitancy

Variables	Strongly disagree	Disagree	Somewhat disagree	Either agree	Disagree	Somewhat agree	Agree
Vaccine Attitude Examination							
Mistrust of Vaccine benefits							
Worries over unforeseen future effects							
Concern about commercial profiteering							
Preference for Natural Immunity							
Psychological antecedents to vaccination	Strongly disagree	disagree	somewhat disagree	either agree	disagree	somewhat agree	agree
Confidence							
Complacency							
Constraints							
Calculation							
Collective responsibility							
Perceived sensitivity to vaccine	Strongly disagree	disagree	somewhat disagree	either agree	disagree	somewhat agree	agree
My body is very sensitive to medicines.							
My body overreacts to medicines.							
I usually have stronger reactions to medicines than most people.							
I have had a bad reaction to medicines in the past.							
Even very small amounts of medicines can upset my body							
Total score							

**Table 9:** Characteristics of the respondents

Variables	
Sex	
Male	
Female	
Age groups (years)	
60-64 years	
65-69 years	
70-74 years	
75-79 years	
80 years and above	
Marital Status	
Married	
Widowed	
Separated	
Highest Educational attainment	
No formal	
Primary	
Secondary	
Tertiary	
Living arrangement	
Alone	
With spouse only	
With children/ grandchildren	
With relatives and friends	
Financial support	
Self-supporting	
Spouse only	
Children/ grandchildren	
Relative and friend	
Social support	
Self-supporting	
Spouse only	
Children/grandchildren	
Relative and friend	
Source of health treatment in the past one year	
Clinic	
Chemist	
Traditional settings	
Self-medication	
Self-rated physical activity	
Not active	
Moderately active	
Very active	

**Table 10:** Vaccine hesitancy

Questions	YES	NO
Have you had COVID-19 vaccines?		
Would you like to take the COVID-19 vaccine if was available now?		

**Table 11:** Sources of information on the COVID-19 vaccine hesitancy

Sources of information		
		Social media	Yes	
No		
Television	Yes	
	No	
Radio	Yes	
	No	
Printed and electronic newspapers	Yes	
	No	
Face-to-face communication	Yes	
	No	
Official government website	Yes	
	No	
